# Maize IgE binding proteins: each plant a different profile?

**DOI:** 10.1186/1477-5956-12-17

**Published:** 2014-03-20

**Authors:** Cátia Fonseca, Sébastien Planchon, Carla Pinheiro, Jenny Renaut, Cândido Pinto Ricardo, M Margarida Oliveira, Rita Batista

**Affiliations:** 1 National Health Institute Dr. Ricardo Jorge, Av. Padre Cruz, 1649-016 Lisboa, Portugal; 2 Centre de Recherche Public - Gabriel Lippmann, Department of Environment and Agrobiotechnologies (EVA), Proteomics Platform 41, rue du Brill, L-4422 Belvaux, Luxembourg; 3 Instituto de Tecnologia Química e Biológica, Universidade Nova de Lisboa, Av. da República, 2780-157 Oeiras, Portugal; 4 IBET, Apartado 12, 2781-901 Oeiras, Portugal

**Keywords:** Maize allergens, Western-blot, 2D-gel electrophoresis, Maize proteins

## Abstract

**Background:**

Allergies are nearly always triggered by protein molecules and the majority of individuals with documented immunologic reactions to foods exhibit IgE hypersensitivity reactions. In this study we aimed to understand if natural differences, at proteomic level, between maize populations, may induce different IgE binding proteins profiles among maize-allergic individuals. We also intended to deepen our knowledge on maize IgE binding proteins.

**Results:**

In order to accomplish this goal we have used proteomic tools (SDS-PAGE and 2-D gel electrophoresis followed by western blot) and tested plasma IgE reactivity from four maize-allergic individuals against four different protein fractions (albumins, globulins, glutelins and prolamins) of three different maize cultivars. We have observed that maize cultivars have different proteomes that result in different IgE binding proteins profiles when tested against plasma from maize-allergic individuals. We could identify 19 different maize IgE binding proteins, 11 of which were unknown to date. Moreover, we found that most (89.5%) of the 19 identified potential maize allergens could be related to plant stress.

**Conclusions:**

These results lead us to conclude that, within each species, plant allergenic potential varies with genotype. Moreover, considering the stress-related IgE binding proteins identified, we hypothesise that the environment, particularly stress conditions, may alter IgE binding protein profiles of plant components.

## Background

Cereals are the most important crops in the world. For the majority of the human population, cereal-based foods constitute the most important source of energy and several nutrients. In the poorest parts of the world starchy foods, including cereals, may supply 70% of total energy. Although a number of cereal species are grown worldwide for food, only three - maize, wheat and rice (respectively, 872, 671 and 720 million tonnes produced in 2012) - account, together, for over 85% of the total production (FAOSTAT- http://faostat.fao.org/site/567/DesktopDefault.aspx?PageID=567#ancor).

Maize is present in a wide range of foods (bread, breakfast cereals, corn snacks, corn flour, polenta, popcorn, sweat corn). Maize variation may be categorized on the basis of quality, quantity and pattern of kernel endosperm composition. Consequently, maize categories are generally divided in: flint, dent, flour, sweet and pop corn [1].

Maize allergy can occur after the ingestion of maize or maize derivatives, or by the inhalation of maize flour or pollen. Recently, some papers were published on maize allergy [2-4]. However, the factors that may influence allergy elicitation are still unknown.

Considering that proteins are the elicitors of the majority of allergic food reactions, it would be expected that different cultivars may induce different allergic reactions. A high heterogeneity in the distribution and quantification of several already known plant allergens among different cultivars has been reported for peanut, soybean, tomato, maize and apple [5-9]. In this study we aimed to contribute to the characterisation of maize flour IgE binding proteins *via* proteomic tools trying to understand if natural proteomic differences, between maize varieties, may result in different IgE binding proteins profiles among maize-allergic individuals. In order to accomplish this goal we have tested plasma IgE reactivity from four maize-allergic individuals against four different protein fractions (albumins, globulins, prolamins and glutelins) of three different maize cultivars.

## Results

In a recent publication we have compared the potential allergenicity of MON 810 maize against its non-transgenic counterpart [4]. In that work the tested plants were grown for several generations under the same environmental conditions in order to minimize the environmental influence in final differences. We found that the tested individuals reacted very similarly to MON810 *vs*. its control and have identified 14 new maize IgE binding proteins. In the present study, we aimed to better understand the influence that the natural differences of the plant proteomes may have on plant IgE binding proteins profiles. We also intended to contribute to the knowledge of maize IgE binding proteins. In order to assess how plant variability may affect individual IgE binding, we have compared plasma IgE reactivity of four maize-allergic individuals to four protein fractions of three maize cultivars. 2-D gel electrophoresis and western blot with plasma from the same maize-allergic individuals were used, to identify maize IgE binding proteins.

### Each individual is an individual and each variety a variety

**Figure 1:**
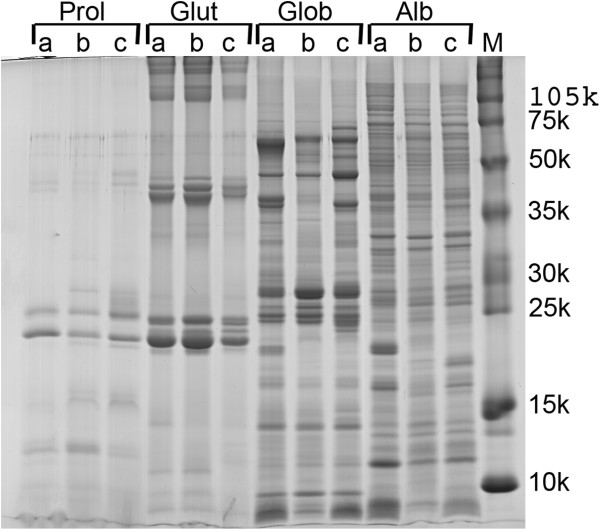
**SDS-PAGE of four protein fractions from three different maize varieties.** Protein fractions: **Prol** (Prolamins), **Glut** (Glutelins), **Glob** (Globulins) and **Alb** (Albumins), (30 μg protein/lane). The maize varieties tested were: **a**- maize commercial line Tietar, **b**- inbred line PB 269 (FLINT type), **c**- inbred line PB 369 (DENT type). **M**- Protein molecular weight marker (10^−3^).

**Figure 2:**
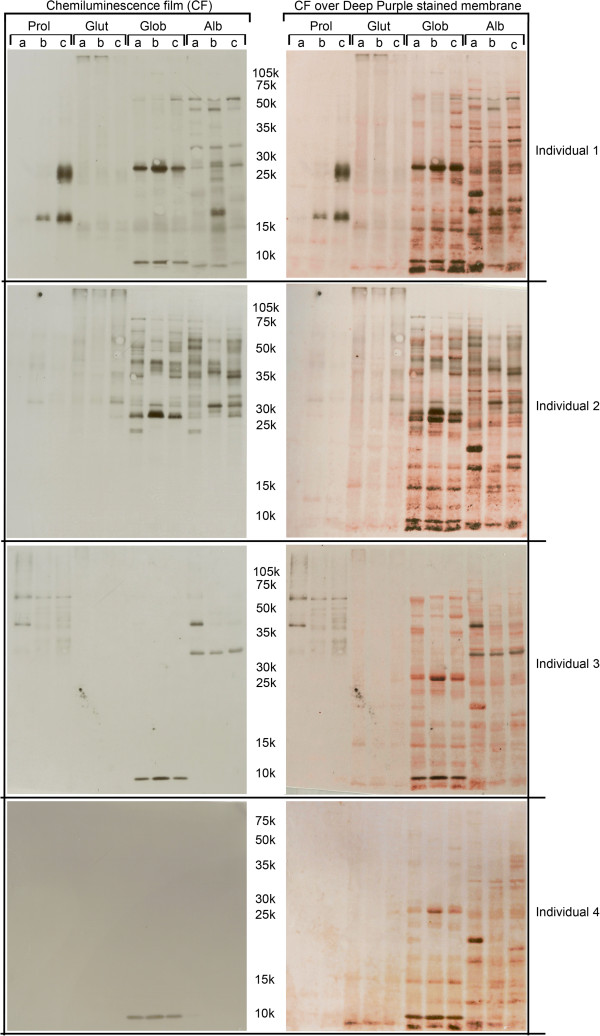
**Western blot with plasmas from four maize-allergic individuals (see Table**1**for information).** Left panel: chemiluminescence films; Right panel: chemiluminescence films over pre-coloured nitrocellulose membranes; Protein fractions: **Prol** (Prolamins), **Glut** (Glutelins), **Glob** (Globulins) and **Alb** (Albumins); Maize tested varieties: **a**- maize commercial line Tietar, **b**- inbred line PB 269 (FLINT type), **c**- inbred line PB 369 (DENT type). Molecular weights (10^−3^) are indicated.

As expected, in this study we clearly saw different SDS-PAGE protein profiles (Figure 1) among the three tested maize varieties. We have also confirmed that not only did different patients react differently to the same maize cultivar, but also that the same individual may react differently to different maize cultivars (Figure 2). This is, for instance, clearly seen in individual 1 Immunoblot against prolamins and albumins protein fractions, and in individual 2 immunoblot against globulins and albumins protein fractions (Figure 2). The control plasma (individual 5, Table 1) showed no reaction against any of the maize varieties under test (data not shown).

**Table 1:** Individuals tested in this study

**Patients**	**Age**	**Sex**	**Maize-specific UniCAP (kU/l)**	**Eczema**	**Allergy symptoms**	**Other reported food allergies**	**Other allergies**
**1**	41	M	30.0 (class 4)	No	Itchy eyes; sneezing; wheezing; hives; intense swelling and vomiting	Nuts, beans or seeds (32.8 kU/l -soybean CAP), milk, fish, meat, eggs, wheat products, some fruits and vegetables.	Penicillin, several animals’ hair (cat, dog, horse, hamster…).
**2**	28	M	16.8 (class 3)	No	Hives; wheezing; peanut cause swelling of the throat.	Peanut, soybean (no exact CAP results, but tested positive for peanut and soybean).	Animals’ hair (cat, dog, horse, pigeon), dust, mildew, insect hypersensivity.
**3**	58	F	9.3 (class 3)	No	Sneezing; wheezing.	Egg white.	House dust, pollens
**4**	29	F	5.0 (class 3)	No	Hives; itchy eyes; sneezing; wheezing.	Wheat products, nuts, beans or seeds (no specification).	Grasses, hay, small reaction to dogs’, cats’ and horses’ hair.
**Control**	6	F	<0.35 (class 0)	--	--	--	--

In this work, we found differences in staining efficiency dependent on the protein fraction. On, SDS-PAGE, Colloidal Coomassie blue caused lower staining efficiency of prolamins as compared with the other protein fractions (Figure 1). We also found that, in nitrocellulose membranes, Deep Purple showed higher efficiencies in staining albumins and globulins, as compared with prolamins and glutelins (Figure 2). Van den Broeck and co-workers [10] had already reported that, due to an atypical amino acid composition, the staining method used to visualize prolamins, in gels, may affect the resulting protein pattern.

### IgE binding proteins

**Figure 3:**
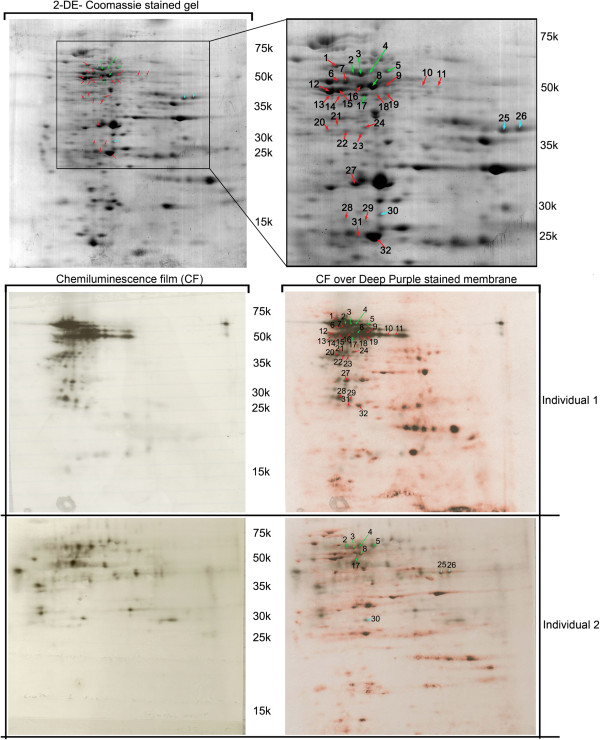
**IgE immunoblot reactivity assay of plasma from maize-allergic individuals (Table**1**for information) against albumins protein fraction. Red arrows** represent immunoreactive spots exclusively detected in individual 1, **blue arrows** represent immunoreactive spots exclusively detected in individual 2 and **green arrows** represent spots detected with plasmas from both patients. Molecular weights (10^−3^) are indicated.

**Figure 4:**
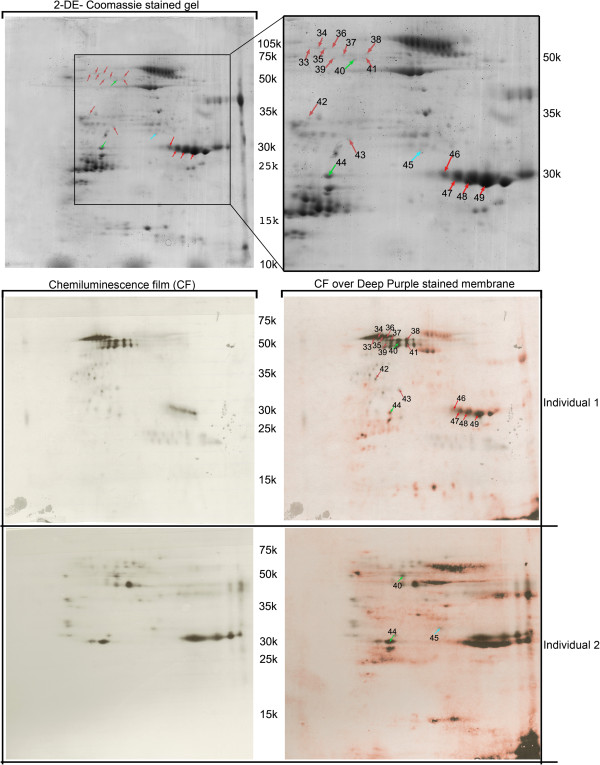
**IgE immunoblot reactivity assay of plasma from maize-allergic individuals (Table**1**for information) against globulins protein fraction. Red arrows** represent immunoreactive spots exclusively detected in individual 1, **blue arrows** represent immunoreactive spots exclusively detected in individual 2 and **green arrows** represent spots detected with plasmas from both patients. Molecular weights (10^−3^) are indicated.

**Figure 5:**
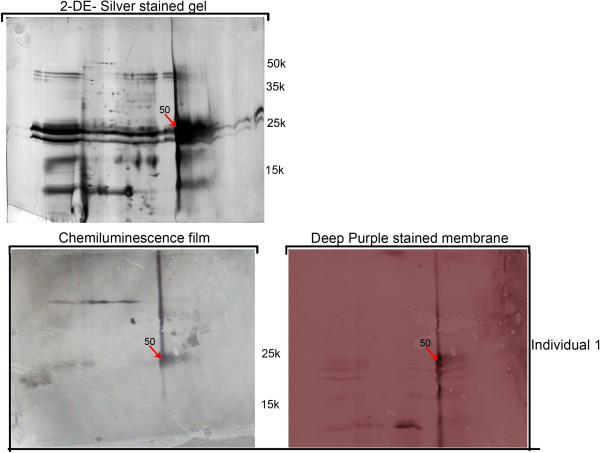
**IgE immunoblot reactivity assay of plasma from maize-allergic individual 1 (Table**1**for information) against prolamins protein fraction.** Molecular weights (10^−3^) are indicated.

For the identification of maize IgE binding proteins, 2-D gel electrophoresis was performed, on each of the four protein fractions from the three maize cultivars, followed by electroblotting and reaction with the most reactive plasmas (see “Gel electrophoresis of maize protein extracts” on Methods section). The use of individuals 1 and 2 plasmas, in this assay, was due to the fact that these were the ones with maize specific IgE concentrations higher than 10 kU/l (an important condition for quality and reliable 2D immunoblot signals). The use of separated protein fractions, as well as Deep-purple stained nitrocellulose membranes, largely facilitated the match between chemiluminescence signals and gel spots and, consequently, the identification of maize IgE binding proteins. The difficulties encountered in the staining of glutelins fraction nitrocellulose membranes, as well as the attainment of low intensity chemiluminescence signals for this protein fraction, made the correlation between chemiluminescence signals and glutelins gel spots an impossible task. High reproducibility between replicates was obtained, both for Deep-purple stained nitrocellulose membranes and immunoblots. We have analysed 50 spots by MS/MS (identified by arrows in Figures 3, 4 and 5) and obtained identification for 38 of them, corresponding to 19 different IgE binding proteins (Table 2 and Additional file 1: Table S1). From these 19 proteins, only 8 had already been identified as maize IgE binding proteins (Table 2) [4]. These were Zea m 22 (an Enolase), Zea m chitinase (a chitinase), Zea m G2 (Globulin-2-precursor), a Ketol-acid reductoisomerase, an UTP-glucose-1-phosphate uridyltransferase, a Fructose-bisphosphate aldolase cytoplasmic isozyme, a Triosephosphate isomerase and a Chaperonin CPN60-1.

**Table 2:** Identified Maize IgE binding proteins

	**Maize allergen**	**Spots Nº**	**Protein information**	**Signal on immunoblot of individual:**	
				**1**	**2**
Already reported maize IgE binding proteins	Zea m 22	8, 9, 12, 15, 16,	*Enolase 48,2 kDa (theor.)*	8,9,12,15,16	8
	Zea m chitinase	49, 50	*Chitinase 29,0 kDa (theor.)*	49,50	___
	Zea m G2	46,47,48	Globulin-2 precursor/Cupin superfamily	46,47,48	___
		5	*Ketol-acid reductoisomerase 63.6 kDa (theor.)*	5	5
		6,7	*UTP-glucose-1-phosphate uridylyltransferase*	6,7	___
		21, 25, 26	*Fructose-bisphosphate aldolase cytoplasmic isozyme*	21	25, 26
		32	Triosephosphate isomerase, cytosolic	32	___
		1	*Chaperonin CPN60-1, mitochondrial precursor*	1	___
New potential maize IgE binding proteins		2,3,4,18,19,22,35	*Granule-bound starch synthase I*	2,3,4, 18,19, 22, 35	2,3,4
		10,11	*Alanine aminotransferase*	10,11	___
		13,14	*Homogentisate 1,2-dioxygenase*	13, 14	___
		17	*rab GDP dissociation inhibitor alpha-like*	17	17
		20	*Glutamine synthetase root isozyme 4*	20	___
		23	*Alpha-1,4-glucan-protein synthase*	23	___
		24	*Protein Z*	24	___
		29	*Lipoprotein*	29	___
		30	*hydroxyacylglutathione hydrolase*	___	30
		31	*Proteasome subunit alpha type 2*	31	___
		27,43	*General stress protein 39*	27, 43	___

*Italic*: Stress related proteins.

Among the identified IgE binding proteins we could scrutinize two main situations: a) IgE binding proteins identified in the immunoblots of only one of the tested individuals (e.g. Chitinase (spots 49, 50), hydroxyacylglutathione hydrolase (spot 30)); b) IgE binding proteins identified in the immunoblots of both individuals (e.g. Ketol-acid reductoisomerase (spot 5), rab GDP dissociation inhibitor alpha-like (spot17)). It is important to notice that some of the IgE binding proteins originated several gel spots (probably due to the existence of different protein isoforms) that, in some cases, selectively bind to the plasma IgE of patient 1, 2, or both. (e. g. Enolase (spots 8, 9, 12, 15, 16), Fructose-bisphosphate aldolase cytoplasmatic isozyme (spots 21, 25, 26)).

#### Identification of maize IgE binding proteins unknown to date

In this study, we have thus identified 11 new maize IgE binding proteins: spots 2, 3, 4, 18, 19, 22 and 35 match a Granule-bound starch synthase I (GBSSI); spot 10 and 11 correspond to Alanine aminotransferase; spots 13 and 14 highest score matches Homogentisate 1,2-dioxygenase; spot 17 matches a rab GDP dissociation inhibitor alpha-like protein; spot 20 corresponds to Glutamine synthase; spot 23 matches alpha-1,4-glucan-protein synthase; spot 24 corresponds to protein Z; spot 29 highest score matches a lipoprotein; spot 30 corresponds to hydroxyacylglutathione hydrolase (Glyoxalase II); spot 31 matches Proteasome subunit alpha type 2; spots 27 and 43 correspond to General stress protein 39.

## Discussion

### Each individual is an individual and each variety a variety

It was already clearly demonstrated that protein profiles can discriminate not only the maize genotype, but also, the geographic location and the season of its cultivation [11]. Thus, the identification of different SDS-PAGE protein profiles for the maize varieties under test was already expected.

In this study we have confirmed that maize cultivar natural proteomic differences may result in different IgE binding profiles when using plasma of a given patient. Panda *et al*. [8] had already reported similar results showing variation in individual qualitative IgE binding to different soybean cultivars. These results lead us to question if allergic symptoms may be dependent on the proteomic level of certain allergens.

Although, as already stated, several authors have documented different allergen content among plant food cultivars [5-9], there is only a small number of studies relating allergen content differences with distinct clinical reaction. However, Carnés *et al*. [12] obtained differences in specific IgE values and SPT wheals when testing apple allergic patients against different apple varieties. Carnés *et al*. also found different SDS- PAGE antigenic profiles among the 10 apple varieties as well as a significant variation in the content of the Mal d 3 allergen.

Also, Dölle *et al*. [13], demonstrated using skin prick tests (SPT), double-blind placebo-controlled food challenges (DBPCFC) and basophil activation test (BAT) the induction of distinct clinical reactivity in tomato-allergic patients, by different tomato cultivars [13].

### Maize IgE binding proteins newly identified may be related to stress response

As already stated above, we have identified 11 new maize IgE binding proteins. Interestingly, all of them may be related to plant stress response. Spots 2, 3, 4, 18, 19, 22 and 35 match a Granule-bound starch synthase I (GBSSI). This is the major enzyme responsible for amylose synthesis and, in rice, it is known to be influenced by drought and temperature [14]. Spot 10 and 11 correspond to Alanine aminotransferase, a protein that is involved in carbon and nitrogen metabolism, converting pyruvate and glutamate to alanine and 2-oxoglutarate. This enzyme is crucial for the rapid conversion of alanine to pyruvate during recovery from low-oxygen stress, in *Arabidopsis thaliana*[15]. Spots 13 and 14 highest score matches Homogentisate 1,2-dioxygenase, an enzyme involved in several pathways, namely aminoacid degradation. The overexpression of this protein was already related to osmotic stress in rice [16]. Spot 17 matches a rab GDP dissociation inhibitor alpha-like protein. These proteins play a central role in the control of vesicle trafficking, and are involved in early stage of the plant’s response to pathogenic attack in rice [17]. Spot 20 corresponds to Glutamine synthase, a key enzyme in plant nitrogen metabolism. This enzyme was already related to salt stress tolerance in rice [18]. Spot 23 matches alpha-1,4-glucan-protein synthase, an enzyme involved in the biosynthesis of cell wall polysaccharides in plants. This protein was already related to chilling injury of peach fruit mesocarp [19] and was also found to be involved in the response of rice roots to cadmium [20]. Spot 24 corresponds to protein Z, a protein that belongs to the serpins superfamily which is a set of proteins able to inhibit proteases. Lampl and co-authors [21] demonstrated that, the major Arabidopsis serpin (AtSerpin1) target protease is the protein Responsive to Desiccation- 21 (RD21). Spot 24 highest score matches a lipoprotein. The influence of stress on lipid metabolism and consequently on lipoprotein particles content is already known [22,23]. Spot 30, corresponds to hydroxyacylglutathione hydrolase (Glyoxalase II) which is part of the glutathione-dependent glyoxalase detoxification system. The overexpression of Glyoxalase II gene was already related to enhanced salinity tolerance in *Brassica juncea*[24]. Spot 31 matches Proteasome subunit alpha type 2, a protein largely related to plant stress response [25]. Finally, spots 27 and 43 correspond to General stress protein 39, a short-chain dehydrogenase/reductase SDR family protein associated, between others metabolic processes, with Abscisic acid (ABA) biosynthesis [26].

Moreover, we found that most (89.5%) of the 19 identified IgE binding proteins could be related to plant stress (Table 2).

### Environmental stimuli may have a crucial role on plant IgE binding proteins profiles

Plant development is regulated by the environment. Moreover, extreme and/or unfavourable conditions impose stresses to which the plant has to respond [27-30], either adapting and restoring its homeostasis, or failing and dying. Plant proteomes are, consequently, constantly changing in response to abiotic and biotic stimuli. Some of these proteomic adjustments may occur due to long-term genomic alterations, others may persist for several generations (for example through epigenetic mechanisms [31]), and others may last only one generation, or even less than that.

The discussion about the potential impacts of plants stress response (and, on a broader perspective, of climate change) on the safety of plant food products is now beginning to emerge. Even though the number of studies is low, some authors have already confirmed that environmental stimuli may have a crucial role in plant allergen expression [32-34].

In this work we have confirmed that there is a high percentage of stress-related proteins among maize IgE binding proteins, which agrees with our previous results [4]. Also relevant, is the fact that among the 11 new maize IgE binding proteins identified, five correspond to proteins already detected as potential allergens in other organisms. One rab GDP dissociation inhibitor was previously identified as a *Aspergillus fumigatus* potential allergen [35]. Protein Z was already identified as a relevant allergen present in beer [36]. A 33 kDa rice allergen was identified as a glyoxalase [37]. A proteasome subunit protein was already reported as a *Hevea brasiliensis* latex allergen [38]. Finally, as general stress protein 39, several fungal allergens belong to SDR protein family [35-40].

Since plants are permanently challenged by inevitable environmental stimuli, our results lead us to hypothesise that the IgE binding protein profile of a given plant may, also, change as fast and as transiently/permanently as its proteome.

### Challenges that arise from the potential influence of genotype and environment on plant IgE binding protein profiles

Although scientific data support the idea that genotypes and environmental conditions may influence plant allergomes and consequently individual clinical reactions, most probably these factors won’t be enough to eliminate allergy of a given food plant. This hypothesis would only be possible to be assessed when allergens and thresholds of elicitation could be estimated with adequate certainty [41]. Until then, the possibility of shaping the allergenicity of a given food plant, by choosing the adequate variety and controlled growth conditions, is still a non- accomplished challenge. Consequently, allergic individuals must continue to avoid consumption of foods that elicits theirs reactions.

Another challenge concerns the evaluation of potential allergenicity of genetically modified food plants. While for some, the potential fast changeability of plant allergomes enhances the importance of monitoring natural variation of allergen expression in non-transgenic plants, under different environmental conditions, before assessing the impact of biotechnology transformations on endogenous levels of allergens, others question the relevance of this evaluation [8]. The complexity of some plant allergomes and their full characterization (formal guidelines for endogenous allergens are still lacking), the difficulty in the acquisition of a sufficient number of well characterized allergic serum donors, as well as the cost of such assays are some of the critical issues [8]. Moreover, other issues like what tests provide sufficient information to allow confident safety conclusions still need to be addressed. In fact, since there are insufficient reliable data regarding allergen thresholds of elicitation the current advice to allergic patients is always to avoid the offending food and products which contain or may contain it. In addition there are no reliable data relating differences in allergenic levels in different crops varieties, with the sensitization process. This together with the fact that consumers most probably do not eat only one variety but a mixture grown at numerous locations, contributes to aggrandize the discussion about the relevance on measuring endogenous allergens in the first place.

## Conclusions

This work contributes to the characterization of maize IgE binding proteins and emphasizes the idea that plant allergomes are not static, but potentially influenced by the plant genotype and growth conditions.

## Methods

### Plant materials

The tested maize varieties included two inbred lines (PB369 -DENT type- and PB 269 -FLINT type) stored at the Portuguese Plant Germoplasm Bank (Braga) and one maize commercial variety (Tietar).

The inbred lines were multiplied during the Spring of 2007 in the same field of “Escola Superior Agrária de Coimbra” (ESAC, Coimbra, Portugal) under controlled pollination. Commercial variety plants were self-pollinated and grown from May to October in a mixture of turf and soil (1:1) in a glass house of “Instituto de Tecnologia Química e Biológica (ITQB, Oeiras, Portugal).

Seeds were randomly collected from three different ears/plants of each line (triplicates), ground in a water mill and sieved through a 150 μm mesh, before protein extraction (9 samples = 3 independent flour samples × 3 maize lines).

### Plasma samples

Plasmas were purchased from Plasmalab International (Everett, WA, USA) and obtained from 4 individuals who had a positive history of documented maize-allergy as well as positive specific UniCAP test values equal or higher than class 3 (Pharmacia Diagnostics) (individuals 1–4, Table 1). Plasma from a non-allergic individual with a class 0 UniCAP test was used as negative control (individual 5, Table 1).

### Maize protein extractions

Maize seed proteins were sequentially extracted with water (albumins), 5% (w/v) NaCl (globulins), 75% (v/v) ethanol (Osborne prolamins) and 0.25% (w/v) NaOH (Osborne glutelins), as previously described [42].

The supernatants were dialyzed overnight (except ethanol fraction) using SnakeSkin pleated dialysis tubes with a cutoff of 3.5 kDa (Thermo Scientific). Ethanol fraction and all the other dialyzed protein fractions were subsequently lyophilized.

For gel electrophoresis the obtained pellets were dissolved in solubilisation buffer (2 M thiourea, 0.4% (v/v) triton X-100, 7 M urea, 4% (w/v) CHAPS, 1% IPG buffer 3–11, 60 mM DTT) and protein concentration measured according to Ramagli [43], with albumin from chicken egg white (Sigma) as standard.

At the end of this step we had 36 protein pellets (4 protein fractions × 3 ears/ plants × 3 maize lines) (Additional file 2: Figure S1).

### Gel electrophoresis of maize protein extracts

At this stage, protein fraction triplicates (independent sequential extractions of three different plants/ears) were mixed, so that for each maize line we worked with 4 different protein fractions (albumins, globulins, prolamins and glutelins) (12 samples total) (Figure 1).

This work included two gel electrophoresis approaches, one aiming to evaluate if different maize cultivars induce different IgE reactivity (a), and another to increase the knowledge of maize IgE binding proteins (b). In strategy (a) we used Unidimensional SDS-PAGE (sodium dodecyl sulfate-polyacrylamide gel electrophoresis) of the four protein fractions, of the three maize cultivars, followed by immunoblot with plasmas from the four maize-allergic individuals.

For (b), 2-D gel electrophoresis was performed on mixed protein extracts (e.g. albumins extracts from different cultivars were mixed, globulins extracts from different cultivars were mixed…- same protein quantity from each cultivar) and followed by immunoblot with plasmas from the most reactive individuals in unidimensional SDS-PAGE assay.

For unidimensional SDS-PAGE, solubilised pellets were diluted 1: 1 in sample buffer (0.125 M Tris–HCl, 4% SDS, 20% v/v glycerol, 0.2 M DTT, 0.02% bromophenol blue, pH 6.8) and boiled for 5 min before electrophoresis on a 1.5 mm-thick, 12.5% T, 3.3% C acrylamide gel with 3.75% T, 3.3% C stacking gel (30 μg protein/lane).

Regarding 2-D gel electrophoresis, isoelectric focusing was performed on 13 cm long immobilized pH gradient (IPG) strips (Amersham Biosciences) with a non-linear pH gradient range of 3 to 11 in an IPGPhor instrument (Amersham Biosciences). The strips were rehydrated with 400 μg of total protein, for 12 h at 30 V, in solubilisation buffer diluted in 8 M urea, 4% (w/v) CHAPS, 0.5% (v/v) IPG buffer 3–11, and 60 mM DTT, to a final volume of 250 μl. After rehydration, IPG strips focusing, equilibration and SDS-PAGE on 1 mm thick, 12.5% T, 3.3% C gels, were performed as previously described [44].

The gels were run at 15°C with a 15 mA/gel constant current for 15 min, and then at 30 mA/gel. Coomassie Colloidal Blue [45] or MS compatible silver staining [46] was used, for gel staining.

### IgE Immunoblot reactivity assay of plasma from maize-allergic patients

IgE reactivity, against maize samples, was probed after unidimensional, or 2-D gel electrophoresis followed by protein transfer onto Hybond ECL nitrocellulose membranes (Amersham Biosciences). Protein transfer was achieved, at 4°C, by wet transfer in 25 mM Tris, 192 mM glycine, 0.1% SDS, 20% methanol, overnight, at 20 V.

Pre-coloured Deep Purple protein Dye nitrocellulose membranes were used to visualize total protein profiles and allow perfect overlaying of the chemiluminescence film. This procedure allowed us to accurately correlate each chemiluminescent signal with the respective spot/ band on the protein gels.

Blots were blocked at 4°C, overnight, with PBS-T (58 mM Na_2_HPO_4_, 17 mM NaH_2_PO_4_.H_2_O, 68 mM NaCl, 0.2% Tween 20) and 5% skimmed milk powder, and western blot procedures were performed as already described [44].

Blots were visualized after exposure to a high performance chemiluminescence Hyperfilm ECL (Amersham Biosciences). For optimal signal intensity, the exposures were between 5 and 20 min. For each tested individual, and protein extract, 2 immunoblots were obtained. The immunoreactive spots were manually excised from colloidal Coomassie blue or MS compatible silver stained 2-D gels (prolamins) and MS characterized.

### Digestion of 2-D gel spots and MS analysis

Spots of interest have been automatically digested and spotted with the Ettan Spot Handling Workstation (GE Healthcare). After washing and desalting in 50 mM ammonium bicarbonate/50% (v/v) methanol, followed by 75% v/v acetonitrile, spots were digested with 40 ng Trypsin Gold for 6 h at 37°C (MS grade, Promega, 5 μg.mL^−1^ in 20 mM ammonium bicarbonate). After extraction, the dried peptides, dissolved in 50% acetonitrile/0.1% trifluoroacetic acid, were spotted on disposable target plates (ABsciex) prior to the deposit of the matrix (α-Cyano-4-hydroxycinnamic acid 7 mg.mL^−1^ in 50% v/v acetonitrile, 0.1% v/v trifluoroacetic acid (biosolve)).

### Database search

Peptide mass determinations were carried out using the 5800 Proteomics Analyzer (ABsciex) in reflectron mode for both peptide mass fingerprint and MS/MS. Calibration was performed with the peptide mass calibration kit for 4700 (ABsciex). Protein identification was done by searching the MS and MS/MS data against NCBI database, downloaded on June 04, 2012, in the Viridiplantae taxonomy (1078293 sequences), using an in house MASCOT 2.3 server (http://www.matrixscience.com). Two trypsin missed cleavages, four dynamic modifications (methionine and tryptophan oxidation, tryptophan dioxydation and tryptophan to kynurenin), and carbamidomethylation of cysteine as fixed modification were allowed. Mass accuracy was set to 150 ppm for parent ions and 0.75 Da for MS/MS fragments. Homology identification was retained with probability set at 95%. All identifications were confirmed manually.

## Abbreviations

CHAPS: 3-[(3-cholamidopropyl)dimethylammonio]-1-propanesulfonate; DTT: Dithiothreitol; FAOSTAT: Statistics Division of Food and Agriculture Organization of the United Nations; GDP: Guanosine triphosphate; IgE: Imunnoglubulin E; IPG: Immobilized ph gradient; MS: Mass spectrometry; SDR: Short-chain dehydrogenase/reductase; SDS-PAGE: Sodium dodecyl sulfate - polyacrylamide gel electrophoresis; UTP: Uridine triphosphate.

## Competing interests

All authors declare that there are no competing interests.

## Authors’ contributions

The authors warrant that the article is original, does not infringe upon any copyright or other proprietary right of any third part, is not under consideration by another journal, and has not been previously published. The authors confirm that they have reviewed and approved the final version of the manuscript and concur with submission. CF contributed to the study design; IgE immunoblot reactivity assay of plasma from maize-allergic subjects data acquisition its analysis and interpretation; MS data interpretation; writing and revision the final version of the paper; final approval of the version to be published. SP contributed to MS data acquisition its analysis and interpretation; writing and revision the final version of the paper; final approval of the version to be published. CP contributed to the study conception and design; protein extraction; revision the final version of the paper; final approval of the version to be published. JR contributed to MS data acquisition its analysis and interpretation; writing and revision the final version of the paper; final approval of the version to be published. CPR contributed to the study conception and design; writing and revision the final version of the paper; final approval of the version to be published. MMO contributed to the study conception and design; writing and revision the final version of the paper; final approval of the version to be published. RB contributed to the study conception and design; IgE Immunoblot Reactivity Assay of Plasma from Maize-Allergic Patients data acquisition its analysis and interpretation; MS data interpretation; writing and revision the final version of the paper; final approval of the version to be published. All authors read and approved the final manuscript.

## Supplementary Material

Additional file 1: Table S1 MS results. Click here for file

Additional file 2: Figure S1 SDS-PAGE (10% T and 3,3% C) of three varieties and four extracts (three replicates per variety and extract). Click here for file
