# Supplementary material for: Maize IgE binding proteins: each plant a different profile?
Source: Proteome Sci. 2014 Mar 20;12:17. doi: 10.1186/1477-5956-12-17 (PMC3999935; doi:10.1186/1477-5956-12-17)
Supplement: Additional file 2: Figure S1 — SDS-PAGE (10% T and 3,3% C) of three varieties and four extracts (three replicates per variety and extract). [file 1477-5956-12-17-S2.pdf]

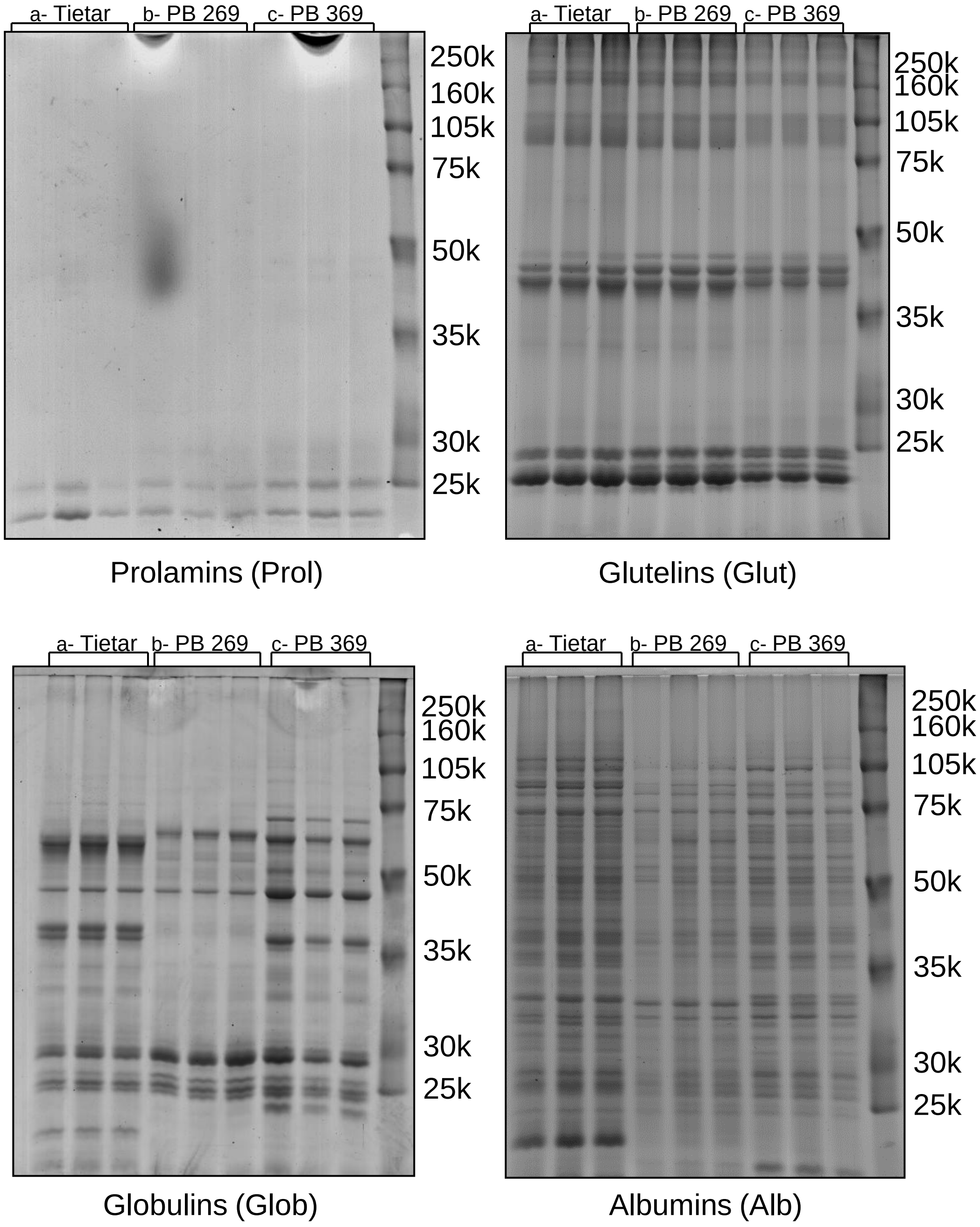


**Fig S1-** SDS-PAGE (10% T and 3,3% C) of three varieties and four extracts (three replicates per variety and extract)
